# Avoidant/resistant rather than tolerant olive rootstocks are more effective in controlling Verticillium wilt

**DOI:** 10.3389/fpls.2022.1032489

**Published:** 2022-10-17

**Authors:** Pablo Díaz-Rueda, Procopio Peinado-Torrubia, Francisco J. Durán-Gutiérrez, Pilar Alcántara-Romano, Ana Aguado, Nieves Capote, José M. Colmenero-Flores

**Affiliations:** ^1^ Plant Ion and Water Regulation Group, Instituto de Recursos Naturales y Agrobiología de Sevilla (IRNAS, CSIC), Sevilla, Spain; ^2^ Andalusian Institute of Agricultural and Fisheries Research and Training (IFAPA) Center Las Torres, Seville, Spain

**Keywords:** graft, *Olea europaea*, qPCR, *Verticillium dahliae* (Kleb), resistance, tolerance

## Abstract

The identification of rootstocks of low susceptibility to *Verticillium dahliae* can become a valuable procedure to achieve effective control of Verticillium wilt in the olive grove. This not only involves the identification of suitable genotypes, but also the study of the interaction between the rootstock and the grafted scion. Thus, a rootstock that prevents or minimizes *V. dahliae* proliferation (avoidance/resistance strategy) can have very different effects on a susceptible scion compared to a rootstock that shows few or no symptoms despite being infected (tolerance strategy). Both resistance and tolerance mechanisms have been recently identified in wild olive genotypes with low susceptibility to *V. dahliae*. When used as rootstocks of the highly susceptible variety ‘Picual’, we found that resistant genotypes, including the cultivar ‘Frantoio’, were more effective than tolerant genotypes in controlling Verticillium wilt. Furthermore, tolerant genotypes were as ineffective as susceptible or extremely susceptible genotypes in controlling Verticillium wilt. We also identified rootstock-scion combinations with behaviours that were not expected according to the degree of susceptibility previously observed in the non-grafted rootstock. Although the rootstocks were able to control Verticillium wilt according to its degree of susceptibility to *V. dahliae*, the ability to control the infection was not adequately transferred to the grafted scion. Our results confirmed that: the degree of susceptibility to Verticillium wilt of an olive variety does not predict its performance as a rootstock; to use a very low susceptible genotype as rootstock of a susceptible scion increases the susceptibility of the genotype used as rootstock; in any case, avoidant/resistant rootstocks are more effective than tolerant rootstocks in reducing the susceptibility of the grafted plant to *V. dahliae*.

## Introduction

The olive (*Olea europaea* subsp. *europaea* L.) crop has enormous economic, cultural and ecological importance within the Mediterranean basin (FAO, 2020). Spain is the main producer and exporter of olive oil worldwide (International Olive Council database, 2022). In recent years, olive cultivation has undergone a green revolution based on the change from a traditional model to a high-density plantation system (Connor et al., 2014). In addition, the use of drip irrigation, the application of forced agronomic practices (high input of fertilization, specially nitrogen, and tillage practices) and the use of young and highly productive cultivars have managed to increase production (Gucci et al., 2007) and yield (Lodolini et al., 2016; Marra et al., 2016). This new scenario, with high soil humidity and the proximity of the tree roots, has facilitated, however, the prevalence and spread of soilborne fungi (Rodríguez et al., 2008).

Verticillium wilt of olive is probably the most devastating fungal disease of the olive tree worldwide (López-Escudero and Mercado-Blanco, 2011; Tsror, 2011). In Mediterranean climate areas, this disease is considered the main limiting factor in olive cultivation because it causes high levels of tree mortality and a reduction in productivity and fruit yield (Montes-Osuna and Mercado-Blanco, 2020). The disease is caused by the soilborne hemibiotrophic fungus *Verticillium dahliae* Kleb. This pathogen is characterized by the production of infectious propagules named microsclerotia, which are resistant structures that allow the fungus to persist in the soil for long time (up to 14 years) in the absence of a host (Wilhelm, 1955; López-Escudero and Mercado-Blanco, 2011; Jiménez-Díaz et al., 2012; Montes-Osuna and Mercado-Blanco, 2020). Thus, long-lasting perennial crops such as olive trees are continuously exposed to the fungal infection (Tjamos and Jiménez-Díaz, 1998).

*V. dahliae* germinates in response to plant root exudates, penetrates through the roots (Mol, 1995) and colonizes the host’s vascular system, progressively decreasing the conductivity of the xylem due to the occlusion of the vessels by tyloses and gels generated by the plant to compartmentalize the infection (Yadeta and Thomma, 2013). The loss of hydraulic conductivity has negative effects on the plant, causing cavitation or embolism, and eventually plant wilting and death (Inch and Ploetz, 2012; Pouzoulet et al., 2014; Deyett et al., 2019). Disease symptoms mainly depends on fungal virulence, and host susceptibility. The defoliating pathotype (D) of *V. dahliae* causes the most severe symptoms, with young olive trees exhibiting more susceptibility than adult ones, in which the infection generally spreads more slowly (López-Escudero and Mercado-Blanco, 2011).

The control of Verticillium wilt is not easy. Drip irrigation, the use of infected planting material and inappropriate agronomic practices contribute to the spread of infective propagules (Montes-Osuna and Mercado-Blanco, 2020). The adequate management of the irrigation system (Baroudy et al., 2018) and the control of the phytosanitary status of the plant material are essential to control the incidence and severity of the disease. Crop rotation is not adequate due to the wide host range of *V. dahliae* (McCain and Raabe, 1981; Pegg and Brady, 2002) and the persistence of microesclerotia in the soil (Umaerus et al., 1989). The application of thermal treatments such as soil solarization (Tjamos and Paplomatas, 1988), hot air (Morello et al., 2016) or the use of organic amendments to inhibit or reduce the viability of the microsclerotia (Varo-Suárez et al., 2018) help to manage Verticillium wilt of olive. On the other hand, the use of chemical fumigation is not very effective or is not allowed due to environmental concerns (Tsror, 2011). The use of biological control agents based in bacterial strains and not pathogenic fungi within an integrated management, delays the appearance of symptoms, and reduces the incidence and severity of the disease (Mercado-Blanco et al., 2004; Prieto et al., 2009; Papasotiriou et al., 2013; Carrero-Carrón et al., 2016; Varo et al., 2016; Gómez-Lama Cabanás et al., 2018; Mulero-Aparicio et al., 2020).

Due to the presence of asymptomatic infected plants and the constant presence of latent microsclerotia in the soil, among other epidemiological factors, the development of molecular tools for the accurate and sensitive detection of *V. dahliae* is essential to control the silent spread of the disease. The real-time quantitative polymerase chain reaction (qPCR) for the specific detection and quantification of *V. dahliae* in infected tissues has allowed a better understanding of the dynamics of *V. dahliae* infection in infected olive tissues and its relationship with the degree of susceptibility of different olive cultivars (Mercado-Blanco et al., 2003; Markakis et al., 2009; Gramaje et al., 2013; Díaz-Rueda et al., 2021). The use of qPCR detection protocols establishes a preventive control method within an integrated management strategy (Montes-Osuna and Mercado-Blanco, 2020), allowing an early diagnosis of Verticillium wilt of olive, even when the symptoms are not evident.

The genetic resistance of the cultivar is the most recommended measure to control Verticillium wilt of olive (Trapero et al., 2015). However, most of the agronomically and economically relevant olive cultivars, such as ‘Picual’ or ‘Arbequina’, are susceptible or extremely susceptible to the defoliant pathotype of *V. dahliae* (López-Escudero and Mercado-Blanco, 2011). On the contrary, a high level of resistance has been found in cultivars such as ‘Empeltre’, ‘Frantoio’, ‘Oblonga’ and ‘Changlot Real’. These genotypes have shown a late onset of the disease, ability to recover and a low percentage of dead plants under *V. dahliae* pressure (López-Escudero et al., 2004; López-Escudero and Mercado-Blanco, 2011). However, none of these resistant cultivars are widely used in commercial olive production due to their poor agronomic properties (López-Escudero and Mercado-Blanco, 2011). Studies on resistance to Verticillium wilt of olive have mainly focused on the analysis of biochemical and physiological responses (Gharbi et al., 2016; Gharbi et al., 2017; Trapero et al., 2018) or on genetic and transcriptomic analyses (Gómez-Lama Cabanás et al., 2015; Leyva-Pérez et al., 2018; Serrano et al., 2020) of olive cultivars that show different levels of susceptibility to *V. dahliae*. However, the genetic origin of resistance in olive trees has not been accurately identified yet.

Grafting susceptible olive cultivars onto resistant rootstocks is an approach that has recently gained interest as a control strategy for Verticillium wilt. The appearance of diseases caused by soil pathogens such as *Verticillium* (Montes-Osuna and Mercado-Blanco, 2020) or *Phytophthora* (González et al., 2019) and the need to obtain dwarfing varieties in super-intensive or hedge cultivation systems (Rallo, 2014) has increased the number of studies on the use of rootstocks in the olive grove. Numerous studies report the use of rootstocks to solve the problem of Verticillium wilt in different herbaceous species such as watermelon (Attavar et al., 2020; Devi et al., 2021), and tomato (Papadaki et al., 2017), and in woody plants such as avocado (Haberman et al., 2020) and pistachio (Epstein et al., 2004). In olive, the grafting strategy has been studied under controlled conditions (Porras Soriano et al., 2003; Bubici and Cirulli, 2012) and in field trials with naturally infested soils (Hartmann et al., 1971; Valverde et al., 2021). The use of resistant rootstocks has been shown to reduce the susceptibility to *V. dahliae*, delaying the onset of symptoms and therefore the development of the disease (Porras Soriano et al., 2003; Bubici and Cirulli, 2012; Yildiz et al., 2020), although this tolerance could be broken over time in soils with high inoculum density (Valverde et al., 2021). However, to our knowledge, no resistant rootstocks have been currently reported that fully prevents Verticillium infection of the grafted variety. For this reason, identification of new resistant olive rootstocks that can control the disease in soils with low and/or high inoculum density is demanded by the olive sector.

Two different strategies can be distinguished in plants to control the infection of pathogens (Robb, 2007). On the one hand, the mechanism that avoids pathogen proliferation, by which plant defences prevent or limit the colonization of the pathogen, which is commonly referred to by the generic term of resistance. On the other hand, the tolerance mechanism inhibits or reduce the symptoms despite the occurrence of pathogen proliferation. Interestingly, both strategies have been recently identified as mechanisms of avoidance/resistance or tolerance to Verticillium wilt in different wild olive genotypes of the SILVOLIVE collection (Díaz-Rueda et al., 2021). The SILVOLIVE collection comprises an extensive number of wild genotypes belonging to different subspecies of *Olea europaea*, providing a natural source of genetic variability with high potential to be used for cultivar or rootstock breeding.

The objective of this study was to clarify which one of the two mechanisms, avoidance/resistance or tolerance, is more effective to control Verticillium wilt in olive plants through the use of low-susceptibility rootstocks. With this aim, the highly susceptible ‘Picual’ cultivar was grafted onto different wild olive genotypes with very low susceptibility to Verticillium wilt that display the two mechanisms (Díaz-Rueda et al., 2021). Verticillium wilt susceptibility was evaluated by estimating the final mean severity of symptoms (FMS), the relative area under the disease progress curve (RAUDPC), and the percentage of dead plants (PDP) in greenhouse conditions. The content of *V. dahliae* DNA was quantified in the rootstocks and in the grafted ‘Picual’ scions at 35 and 120 days after inoculation (dai) by qPCR. Growth parameters such as plant biomass, accumulated length of the grafted scion, number of nodes and number of stem branching were also measured for all combinations and compared with their corresponding non-inoculated plants. The results showed that avoidant/resistant olive rootstocks, which reduce fungus proliferation, were more efficient in controlling Verticillium wilt than tolerant rootstocks.

## Materials and methods

### Plant material

Plant material consisted on cv. ‘Picual’ grafted onto micropropagated wild olive genotypes one year after their *ex-vitro* acclimatization. The different rootstocks used in this study were selected according to their different levels of resistance to Verticillium wilt, previously characterized by Díaz-Rueda et al. (2021). Six genotypes were identified as low susceptible to Verticillium wilt (GUA3, CEH3, AMK27, ACO15, AMK5 and DHO6A). Among them, GUA3, CEH3, AMK27 as well as the cultivar ‘Frantoio’ showed the Verticillium wilt avoidance/resistance strategy, with low amount of *V. dahliae* in the stem of inoculated plants. The amount of *V. dahliae* in the plant was expressed as mean normalized quantity (MNQ) and was calculated as means ± standard errors of the *V. dahliae*:*cox* ratios (ng *V. dahliae* DNA · ng plant DNA^−1^), estimated by qPCR and previously defined by Garrido et al. (2009). Genotypes showing the avoidance/resistance strategy displayed MNQ values of 0.45·10^-4^ - 3.55·10^-4^, whereas ACO15, AMK5 and DHO6A, showing the Verticillium wilt tolerance strategy, had MNQ values of 1.02·10^-3^ - 1.91·10^-3^ (Díaz-Rueda et al., 2021; **Supplementary Figure S1**). AMK21, with a MNQ value of 9.54·10^-3^ was defined as moderately susceptible to Verticillium wilt, while ACZ10 (MNQ value of 1.23·10^-2^) and GUA8 (MNQ value of 1.93·10^-2^) were defined as extremely susceptible. In addition to the resistant ‘Frantoio’, the cultivar ‘Arbequina’ (MNQ value of 1.59) and ‘Picual’ (MNQ value of 3.13) were described as moderately susceptible and extremely susceptible reference controls, respectively (Díaz-Rueda et al., 2021). Cultivars were obtained from a commercial nursery (**Table 1**). Wild-olive genotypes were micropropagated *in-vitro* from the SILVOLIVE germplasm collection (Díaz-Rueda et al., 2020). Two types of grafting procedures were used: ‘spike’ graft and ‘gusset’ or ‘T’ graft. For the ‘spike’ graft, the rootstock was decapitated 15 cm from the base of the stem and the 5-6 cm shoot of the scion containing four to six buds was inserted in an incision made in the cut end of the rootstock trunk. For the ‘gusset’ graft, a T-shaped cut was made at about 20 cm from the base of the rootstock stem, where a rectangular bark slice of the scion containing two buds was introduced. To assure a close contact and the immobility between the rootstock and the scion, the graft area was tied with plastic film.

**Table 1:** Mean disease parameters assessed in cultivar ‘Picual’ grafted onto wild olive rootstocks inoculated with the defoliating isolate VD117 of *Verticillium dahliae*.

Varieties^a^	R.L^b^	RAUDPC^c^		FMS^d^		PDP^e^	
**ARBEQUINA**	**MS**	**81.61 ± *1.59***	a	**4.00 ± *0.00***	**a**	**100.0 ± *0.00***	**a**
ARB/PIC	MS	68.10 ± *4.22*	ab	4.00 ± *0.00*	a	100.0 ± *0.00*	a
**PICUAL**	**ES**	**66.97 ± *2.27***	**ab**	**4.00 ± *0.00***	**a**	**100.0 ± *0.00***	**a**
PIC/PIC	ES	64.06 ± *2.11*	ab	4.00 ± *0.00*	a	100.0 ± *0.00*	a
ACZ10/PIC	S	63.94 ± *3.45*	ab	4.00 ± *0.00*	a	100.0 ± *0.00*	a
GUA8/PIC	ES	62.04 ± *4.26*	ab	4.00 ± *0.00*	a	100.0 ± *0.00*	a
ACO15/PIC	R	58.82 ± *1.44*	ab	4.00 ± *0.00*	a	100.0 ± *0.00*	a
CEH23/PIC	R	58.66 ± *3.23*	ab	4.00 ± *0.00*	a	100.0 ± *0.00*	a
AMK5/PIC	R	54.56 ± *2.18*	abc	4.00 ± *0.00*	a	100.0 ± *0.00*	a
DHO6A/PIC	R	54.20 ± *4.62*	abc	3.75 ± *0.25*	ab	93.8 ± *6.05*	a
AMK21/PIC	MS	46.41 ± *3.97*	cb	4.00 ± *0.00*	a	100.0 ± *0.00*	a
GUA3/PIC	R	40.18 ± *7.34*	c	2.87 ± *0.42*	b	62.5 ± *12.1*	b
AMK27/PIC	R	36.20 ± *4.52*	c	3.46 ± *0.34*	ab	81.25 ± *9.75*	ab
FRA/PIC	R	18.94 ± *6.56*	d	1.56 ± *0.44*	c	28.57 ± *12.07*	c
**FRANTOIO**	**R**	**07.48 ± *8.40***	**e**	**0.48 ± *0.24***	**d**	**9.09 ± *6.13***	**c**

a ‘Picual’ grafted onto genotypes from the SILVOLIVE collection (Díaz-Rueda et al., 2020).

b RL, Resistance level of each rootstock genotype according to Díaz-Rueda et al. (2021). ES, extremely susceptible; S, susceptible; MS, moderately susceptible; R, resistant.

c RAUDPC, Relative area under the disease progress curve estimated as the percentage with regard to the potential maximum value.

d FMS, Final mean severity of symptoms.

e PDP, Percentage of dead plants. ‘Picual’ grafted onto the reference cultivars ‘Picual’ PIC/PIC (extremely susceptible), ‘Arbequina’ ARB/PIC (moderately susceptible) and ‘Frantoio’ FRA/PIC (resistant) are indicated in blue. Non-grafted ‘Picual’, ‘Arbequina’ and ‘Frantoio’ reference controls are indicated in bold. Per columns, different letters indicate significant differences according to LSD test (*P*=0.05).

The newly grafted plants were maintained in 3-L pots containing sterilized 2:1 silt:peat moss (v:v) under greenhouse conditions for 4 months at 28°C and high relative humidity to promote the union and the growth of the graft. During this phase, plants were fertilized, and the lateral buds of the rootstocks were removed, facilitating graft cicatrization and the development of the scions. After this time, inoculation tests for resistance assessment were carried out.

### Fungal isolate and inoculum production

The defoliating *V. dahliae* pathotype VD-117, obtained from the collection of the Plant Pathology Laboratory of Department of Agronomy, University of Córdoba (Spain) (Blanco-López et al., 1984), was used in the inoculation tests for resistance assessment. Conidial suspensions were prepared by transferring five 8 mm-Potato Dextrose Agar (PDA) discs of actively growing mycelium of VD-117 isolate to flasks containing 100 mL Potato Dextrose Broth (PDB) and incubated at 150 rpm at 24°C in the dark for 7 days. The conidial suspension was filtered through four layers of sterile cheesecloth and adjusted to 1 × 10^7^ conidia/mL with sterile distilled water.

### Inoculation of olive plants

For resistance tests, twenty-four plants were inoculated for each rootstock × scion combination. Plant roots were washed under tap water to remove the substrate. The bare root system of each plant were cut 4-5 times in the secondary roots and dipped in the *V. dahliae* conidial suspension for 15 min. Roots of non-inoculated control plants were subjected to the same treatment, but immersed in PDB:sterile distilled water (1:1, v:v). Plants were individually transplanted into 3-L pots containing sterilized 2:1 silt:peat moss (v:v). The experimental design was complete randomized blocks with four blocks and six plants per block and 5-7 non-inoculated plants for each combination. Moreover, non-grafted ‘Frantoio’, ‘Picual’ and ‘Arbequina’ were used as controls following the same experimental design. Plants were maintained at 24/18°C and 60/40% relative humidity (day/night) in a greenhouse where natural illumination was supplemented with 360 µmol m^-2^ s^-1^ fluorescent lighting to ensure 14-h photoperiod. Plants were watered three times per week and fertilized weekly with Hoagland’s nutrient solution (Hoagland and Arnon, 1950).

### Symptoms assessment

Symptoms were evaluated on each plant at 0, 35, 50, 70, 85, 100 and 120 days after inoculation (dai), following a 0-4 rating scale according to the percentage of *M*aximum *I*ntensity *S*ymptoms (*MIS*) based in the percentage of the plant canopy that was affected: total or partial apoplexy, chlorosis, leaf curl, stunting, leaf and shoot necrosis or defoliation: 0 = 0% *MIS* or no symptoms; 1 = 1-25% *MIS*; 2 = 26-50% *MIS*; 3 = 51-75% *MIS*; 4 = 76-100% *MIS* or dead plants.

At the end of the experiment, the following disease parameters were estimated from these scale values: i) the relative area under the disease progress curve (RAUDPC) was obtained for each plant considering its percentage respect to the maximum value that could be reached in the period of assessment, using the following formula based on Campbell and Madden (1990):


(1) $$RAUDPC=\left[\sum_{i=1}^{n}\left(\frac{S_{i}+S_{i-1}}{2}\right)\Delta t\right]\left[\frac{100}{S_{max}T}\right]$$

where S_i_ = severity of the experimental unit in the observation i; ∆t = the number of days between observations; S_max_ = maximum disease rating (=4); T = experimental period in days (=120); n = number of observations; ii) the final mean severity of symptoms (FMS), calculated according to López-Escudero et al. (2007); iii) the percentage of dead plants (PDP) from the total of inoculated plants.

### Growth parameters

Accumulated length of the grafted scion (including secondary and tertiary branches), number of nodes, and number of stem branching were measured at 0 and 120 dai. The increase of the growth for each combination was calculated as the mean ± the standard error of the difference between the value at 120 dai and 0 dai. The relative increase of the growth parameters was calculated as the ratio between the growth increase in inoculated plants and the growth values of non-inoculated plants.

### Quantification of *Verticillium dahliae* in the scion stem and rootstock by real-time PCR

Quantification of the amount of *V. dahliae* DNA in the inoculated plants was carried out at 35 and 120 dai by qPCR using the primers and TaqMan probe designed by Bilodeau et al. (2012) and following the procedure described by Díaz-Rueda et al. (2021). Results were expressed as means ± standard errors of the *V. dahliae*:*cox* ratios (ng *V. dahliae* DNA · ng plant DNA^–1^), previously defined as mean normalized quantity (MNQ) in Garrido et al. (2009). Eight inoculated plants were used at 35 dai (two inoculated plants/block and four blocks), and 16 inoculated plants at 120 dai (four inoculated plants/block and four blocks). Non-inoculated plants were quantified as negative controls.

Plants were removed from the pots and both the rootstock trunk and the stem of the scion were separated and immediately frozen at - 80°C until analysis. For each rootstock x scion combination, grafted scions and rootstocks from plants from the same experimental block were independently grouped, respectively, in a composite sample, giving rise to four biological replicates per plant tissue (scion stems and rootstock trunks), rootstock/scion combination, and harvesting time. Moreover, the emergence of new shoots from the rootstock stems were also recorded and photographed in inoculated plants. These new shoots were harvested from ‘Frantoio’, GUA3, AMK27, AMK21 and DHO6A genotypes at 120 dai and *V. dahliae* DNA was quantified and compared to the amount detected in the corresponding ‘Picual’ grafted scion.

Samples were ground by a blender (Taurus JB1501, Lleida, Spain) and by a mortar in presence of liquid nitrogen until getting a fine powder. Total DNA was extracted using the Isolate II Plant ADN Kit (Bioline, London UK) following the manufacturers’ instructions. DNA concentration was accurately determined in duplicate measurements by using a fluorescent spectrophotometer (Modulus™ II Microplate Multimode reader, Turner Biosystems, USA). PCR reactions and DNA normalization were carried out according to Díaz-Rueda et al. (2021).

### Statistical analysis

Statistical analyses were performed with the software R (R: A language and environment for statistical computing. R Foundation for Statistical Computing, Vienna, Austria). Data were subjected to the analysis of variance (ANOVA). The means of FMS, RAUDPC, PDP, and DNA content of the rootstock and the scion at 35 and 120 dai were compared between each genotype and the means of the grafted reference controls FRA/PIC, ARB/PIC and PIC/PIC by LSD test at P = 0.05. The percentage data were previously transformed by arcsine (Y/100)^1/2^. Linear regression analyses were performed to estimate relationships between symptomatology parameters (RAUDPC) and the DNA content of the rootstock and stem at 35 and 120 dai, respectively.

## Results

### Development of symptoms and growth parameters in inoculated plants

**Figure 1:**
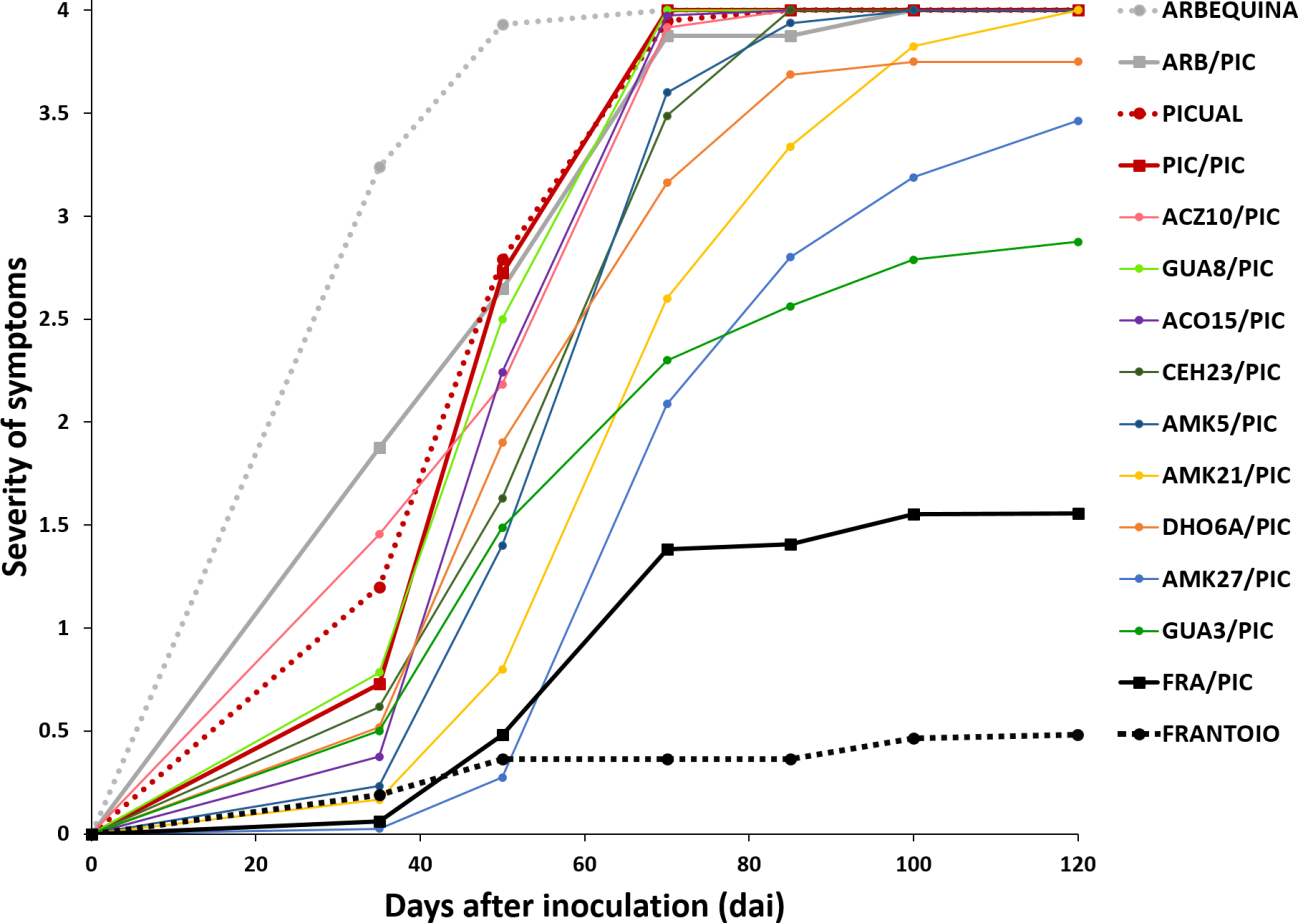
Progress of the severity of symptoms recorded in cultivar ‘Picual’ grafted onto wild olive rootstocks inoculated with the defoliating isolate VD117 of *Verticillium dahliae*. Values are the means of 24 plants. Severity of symptoms was assessed each time on a 0-4 rating scale, according to the percentage of *M*aximum *I*ntensity *S*ymthoms (*MIS*): apoplexy, chlorosis, leaf and shoot necrosis or defoliation: 0 = 0% *MIS* or no symptoms; 1 = 1-25% *MIS*; 2 = 26-50% *MIS*; 3 = 51-75% *MIS*; 4 = 76-100% *MIS* or dead plants. Non-grafted ‘Picual’, ‘Arbequina’ and ‘Frantoio’ reference cultivars are indicated in dashed lines.

**Figure 2:**
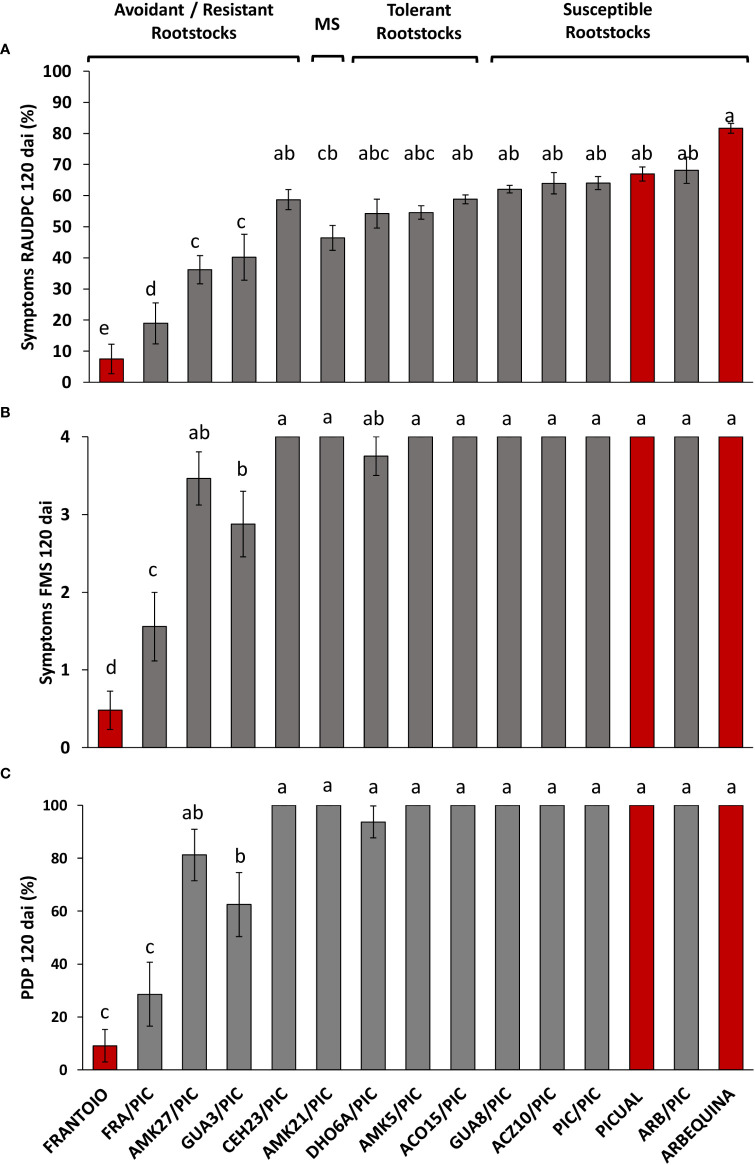
Relative Area Under the Disease Progress Curve (RAUDPC) **(A)**, Final Mean Severity (FMS) **(B)** and Percentage of Dead Plants (PDP) **(C)** of cultivar ‘Picual’ grafted onto different olive rootstocks inoculated with the *Verticillium dahliae* defoliating isolate VD117, at 120 days after inoculation (dai). Red bars correspond to non-grafted reference cultivars: the resistant ‘Frantoio’, the moderately susceptible ‘Arbequina’, and the extremely susceptible ‘Picual’, according to the levels of resistance described by López-Escudero et al. (2007). Results are the mean of 16 plants and error bars correspond to the standard error. Different letters above bars indicate significant differences according to LSD test (P=0.05).

No symptoms were developed in non-inoculated, grafted and non-grafted, plants. The inoculated non-grafted ‘Picual’, ‘Arbequina’ and ‘Frantoio’ cultivars used as reference controls, developed symptoms of Verticillium wilt according to their previously established susceptibility level, except for ‘Arbequina’ that behaved as an extremely susceptible variety in this work (**Table 1** and **Figures 1**, **2**). Thus, 120 dai ‘Picual’ plants presented severe wilting symptoms, showing apoplexy and defoliation in all inoculated plants, with RAUDPC values of 66.97% and FMS values of 4 on the 0-4 symptoms scale (**Table 1**). Although commonly behaving as a moderately susceptible cultivar, ‘Arbequina’ showed the most severe symptoms, including chlorosis, severe defoliation, and leaf and shoot necrosis, with RAUDPC and FMS values of 81.61% and 4, respectively (**Table 1**). The low-susceptibility cultivar ‘Frantoio’ displayed very slight symptoms, showing occasional defoliation or leaf curl, although most of the inoculated plants did not show symptoms. ‘Frantoio’ plants reached RAUDPC and FMS values of 7.48% and 0.48, respectively (**Table 1**). At the end of the experiment (120 dai), the percentage of dead plants (PDP) was 100% for ‘Picual’ and ‘Arbequina’, and 9.1% for ‘Frantoio’ cultivars (**Table 1**).

The reference cultivars and the wild genotypes were used as rootstocks of the susceptible scion ‘Picual’. It should be noted that the type of grafting procedure did not affect the symptomatology of the inoculated plants, showing not significant differences in the development of the disease (**Supplementary Figure S2**). In the short term (35 dai), ‘Picual’ grafted on ‘Frantoio’ (FRA/PIC) on ‘Picual’ (PIC/PIC), and on ‘Arbequina’ (ARB/PIC) tend to show lower symptoms than the corresponding non-grafted rootstocks, although significant differences were only detected in ARB/PIC plants (**Supplementary Figures S3A, B**) This trend changed at the end of the assay (120 dai) in which grafted plants tend to show higher (especially those grafted on ‘Frantoio’ rootstocks) or similar symptoms than non-grafted ones. RAUDPC and FMS values were, respectively: 64.06% and 4 in PIC/PIC; 68.1% and 4 in ARB/PIC; and 18.94% and 1.56 in FRA/PIC (**Table 1**). The non-grafted ‘Frantoio’ displayed the lowest susceptibility at 120 dai, with RAUDPC and FMS values of, 7.5% and 0.5, respectively (**Table 1**; **Supplementary Figures S3C, D**). No PIC/PIC or ARB/PIC plants survived at the end of experiment (120 dai), while the FRA/PIC combination and the ungrafted 'Frantoio' kept respectively 71.4% and 91.9% of the plants alive at the end of experiment. These results confirmed the lower susceptibility to Verticillium wilt of the cultivar ‘Frantoio’ as previously reported, and confirmed its ability to significantly reduce symptoms when used as a rootstock of a susceptible cultivar (**Figure 1**).

The susceptible scion ‘Picual’ was also grafted onto the wild olive genotypes. When grafted onto the extremely susceptible genotypes ACZ10 and GUA8, plants showed the highest RAUDPC values (63.94% and 62.04%, respectively) of the whole set of wild olive genotypes tested (**Table 1** and **Figures 1**, **2**). These susceptible genotypes showed FMS values of 4, and 100% of PDP, not differing significantly from the behaviour exhibited by either the PIC/PIC and ARB/PIC combinations or by the non-grafted ‘Picual’ and ‘Arbequina’ cultivars. Interestingly, ‘Picual’ scions grafted onto the tolerant wild genotypes DHO6A, AMK5 and ACO15, also showed high susceptibility to *V. dahliae*, with RAUDPC values between 58.82% and 54.20% and FMS values of 4 (with the exception of DHO6A, with one surviving symptomless plant 120 dai; **Table 1**). The susceptibility of plants grafted onto the tolerant genotypes was even higher than that of plants grafted onto the moderately susceptible AMK21 (AMK21/PIC), which showed a moderate RAUDPC value of 46.41%, a FMS value of 4, and 100% of PDP.

Except for CEH23, the avoidant/resistant genotypes were the most effective in controlling Verticillium wilt when used as rootstocks of the cultivar ‘Picual’ (**Table 1** and **Figures 1**, **2**). Plants grafted onto GUA3, AMK27 and, especially, the cultivar ‘Frantoio’ showed lower symptoms than plants grafted onto the tolerant genotypes. Thus, GUA3/PIC, AMK27/PIC and FRA/PIC plants showed a delay in the development of the disease exhibiting low RAUDPC values (40.18%, 36.20% and 18.94), moderate symptoms (FMS 2.88, 3.46 and 1.56), and PDP of 62.5% and 81.3% and 28.6%, respectively. GUA3/PIC, AMK27/PIC and FRA/PIC plants that remained alive presented no symptoms at the end of experiment. The lowest susceptibility was achieved by the non-grafted ‘Frantoio’ cultivar. Taken together, these results show that avoidant/resistant genotypes are more effective than tolerant genotypes in controlling Verticillium wilt (**Figure 2**). Despite being the non-grafted CEH23 genotype very efficient in preventing the proliferation of *V. dahliae* (**Supplementary Figure S1**; Díaz-Rueda et al., 2020), it behaved as susceptible when grafted with ‘Picual’ (**Table 1** and **Figure 1**).

**Figure 3:**
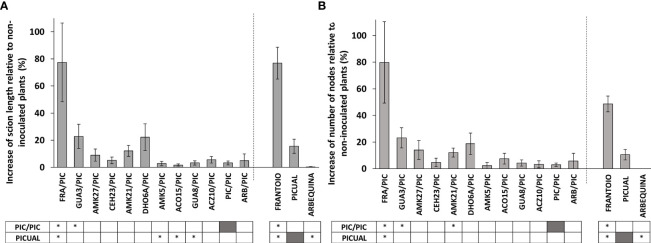
Percentage of increase of scion length **(A)** and number of nodes **(B)** of *Verticillium dahliae*-inoculated ‘Picual’ x rootstock combinations at 120 days after inoculation (dai), relative to non-inoculated plants. Asterisks indicate significant differences with the inoculated self-grafted ‘Picual’ (PIC/PIC) and non-grafted ‘Picual’ plants, respectively. Results are the mean of 12-16 inoculated plants and 3-6 non-inoculated plants. Error bars correspond to the standard error.

Inoculated grafted plants exhibited lower growth than non-inoculated grafted plants at 120 dai (**Supplementary Figures S4A, B**), but plant growth was less inhibited when the ‘Picual’ scion was grafted onto the avoidant/resistant genotypes (**Figure 3**; **Supplementary Figures S4A, B**). Particularly, the avoidant/resistant rootstocks ‘Frantoio’, and GUA3 showed the highest increase of scion length and number of nodes relative to non-inoculated plants.

### Quantification of *Verticillium dahliae* DNA by real-time PCR

**Figure 4:**
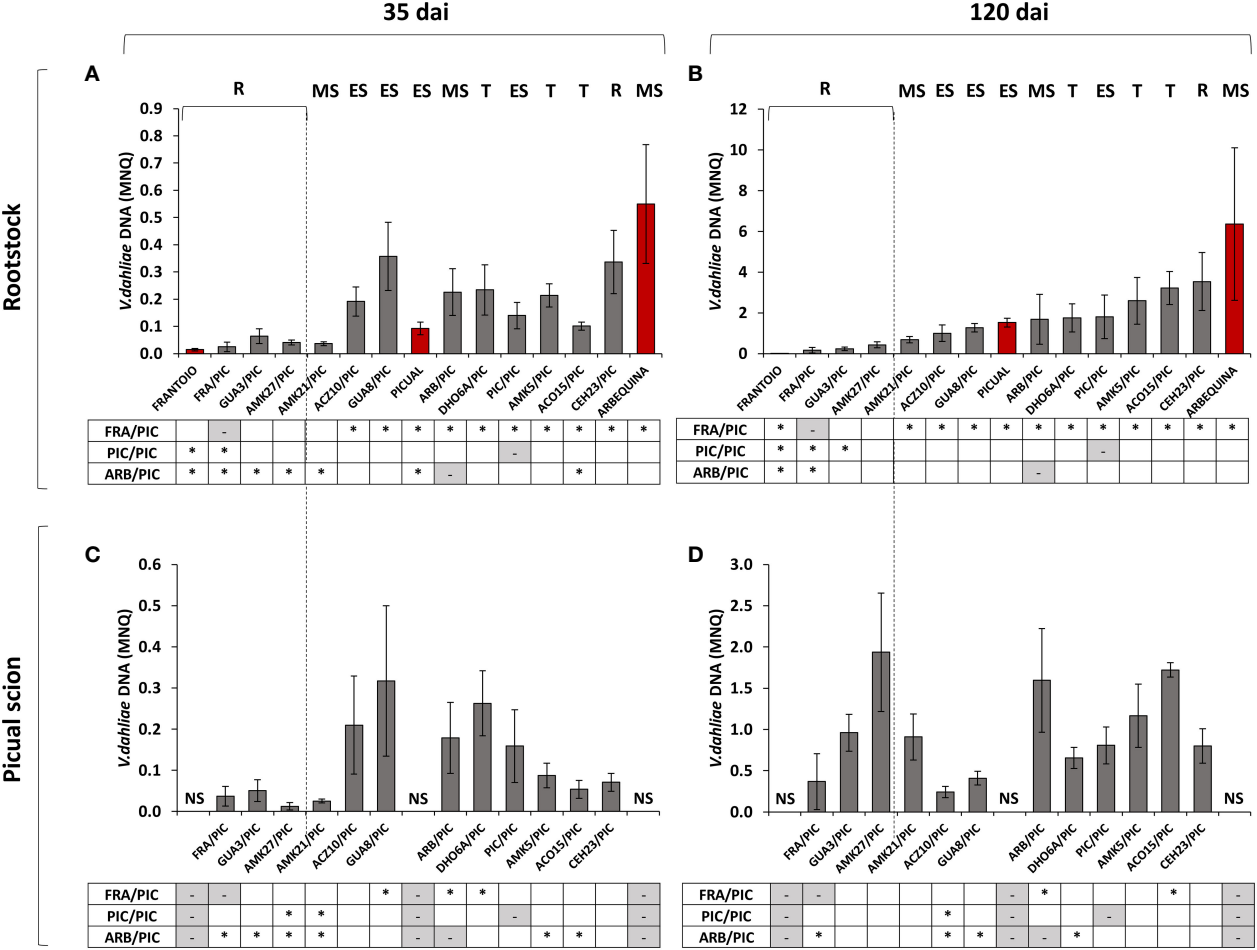
Mean Normalized Quantity (MNQ) of *Verticillium dahliae* DNA in the stem of grafted olive rootstocks evaluated at 35 days after inoculation (dai) with the defoliating isolate VD117 **(A)** and at 120 dai **(B)**. MNQ of *V. dahliae* DNA in the ‘Picual’ grafted scion evaluated at 35 dai **(C)** and at 120 dai **(D)**. Red bars represent non-grafted ‘Frantoio’, ‘Arbequina’, and ‘Picual’ plants as reference cultivars of resistant, moderately susceptible, and extremely susceptible genotypes, respectively. Asterisks indicate significant differences with the DNA values of ‘Picual’ grafted onto ‘Frantoio’ (FRA/PIC), ‘Arbequina’ (ARB/PIC), and ‘Picual’ (PIC/PIC) plants, respectively. NS means No Scion in non-grafted plants. Results are the mean of 16 plants per genotype and error bars correspond to the standard error.

After root inoculation with *V. dahliae*, the degree of infection (fungal proliferation) was measured through direct quantification of *V. dahliae* DNA content in aerial plant tissues. The fungal DNA was quantified in the stem of both the rootstock and the ‘Picual’ scion, below and above the graft site, respectively, at 35 and 120 dai (**Figure 4**).

The ungrafted cultivar ‘Frantoio’ showed the lowest content of *V. dahliae* DNA at both 35 and 120 dai (**Figures 4A, B**). In grafted plants, the avoidant/resistant rootstocks (FRA/PIC, AMK27/PIC and GUA3/PIC) had lower amounts of *V. dahliae* DNA than tolerant and susceptible rootstocks at 35 and 120 dai (**Figures 4A, B**), accordingly to the symptoms previously observed. Tolerant grafted rootstocks (DHO6A/PIC, AMK5/PIC, ACO15/PIC) and susceptible grafted rootstocks (GUA8/PIC, ACZ10/PIC, PIC/PIC) showed statistically significant higher levels of *V. dahliae* DNA in the stem than FRA/PIC at 120 dai (**Figure 4B**). Two wild genotypes showed anomalous performance as rootstocks relative to their behavior as ungrafted plants (Díaz-Rueda et al., 2021), in close correlation with the symptoms exhibited (**Table 1**). On the one hand, the moderately susceptible genotype AMK21 behaved like a resistant rootstock, showing low *V. dahliae* DNA levels in the rootstock stem of the grafted AMK21/PIC plant at 35 dai and 120 dai (**Figures 4A, B**). On the other hand, the resistant genotype CEH23 behaved as a susceptible rootstock, showing high *V. dahliae* DNA levels in the rootstock stem of the grafted CEH23/PIC plant at 35 dai and 120 dai (**Figures 4A, B**).

At 35 dai, ‘Picual’ scions grafted onto the avoidant/resistant genotypes FRA/PIC, AMK27/PIC and GUA3/PIC and also on the moderately susceptible genotype AMK21/PIC had lower *V. dahliae* DNA levels than those grafted on tolerant and susceptible genotypes (**Figure 4C**). These differences were statistically significant in comparison to the susceptible ARB/PIC plants, but only AMK27/PIC and AMK21/PIC showed significantly lower *V. dahliae* DNA levels in comparison to PIC/PIC. However, at 120 dai, a significant increase in fungal DNA was quantified in the stem of the susceptible scion ‘Picual’, even in plants grafted onto resistant genotypes (**Figure 4D**). Thus, the DNA content detected in ‘Picual’ scions grafted onto the resistant and tolerant wild genotypes did not significantly differ from that quantified in PIC/PIC plants at 120 dai.

**Figure 5:**
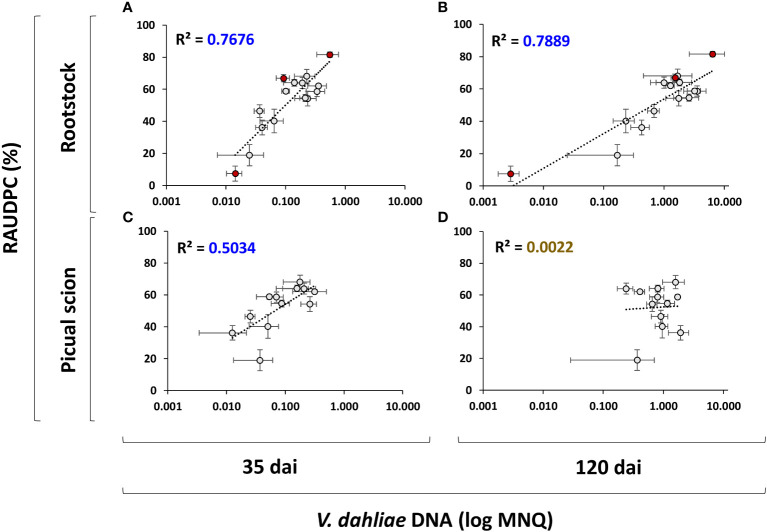
Scatter plots including regression line and coefficient of determination (R^2^) days after inculation (dai) for the relationships between the RAUDPC and the Mean Normalized Quantity (log MNQ) of *Verticillium dahliae* DNA in rootstock trunks at 35 dai **(A)** and 120 dai **(B)**, and in the 'Picual' scions at days after inculation 35 dai **(C)** and 120 dai **(D)**. Results are the mean of 16 plants. Red points indicate non-grafted ‘Frantoio’, ‘Arbequina’ and ‘Picual’ plants. Error bars in two dimensions indicate the standard error of the mean.

The content of *V. dahliae* DNA in the stem of the ‘Picual’ scion grafted onto the avoidant/resistant genotypes at 120 dai (around 0.3 - 1.9 MNQ) was abnormally high if compared to the values previously quantified in the stem of the corresponding ungrafted genotypes (around 0.1·10^-3^ - 0.5·10^-3^ MNQ; **Supplementary Figure S1**). To ascertain which correlation exists between *V. dahliae* DNA content in the rootstock or the scion and the plant symptoms, scatter plots between the RAUDPC and MNQ values were obtained (**Figure 5**).

Results indicated that the rootstock is able to control Verticillium wilt according to its degree of susceptibility to *V. dahliae*, as shown by the high correlation between the amount of *V. dahliae* DNA in the rootstock and the plant symptoms (RAUDPC) at both 35 dai (R^2^ = 0.7676; **Figure 5A**) and 120 dai (R^2^ = 0.7889; **Figure 5B**). However, the ability to control the infection was not adequately transferred to the grafted scion, which showed a worse correlation at 35 dai (R^2^ = 0.5034; **Figure 5C**) and a complete loss of correlation at 120 dai (R^2^ = 0.0022; **Figure 5D**).

**Figure 6:**
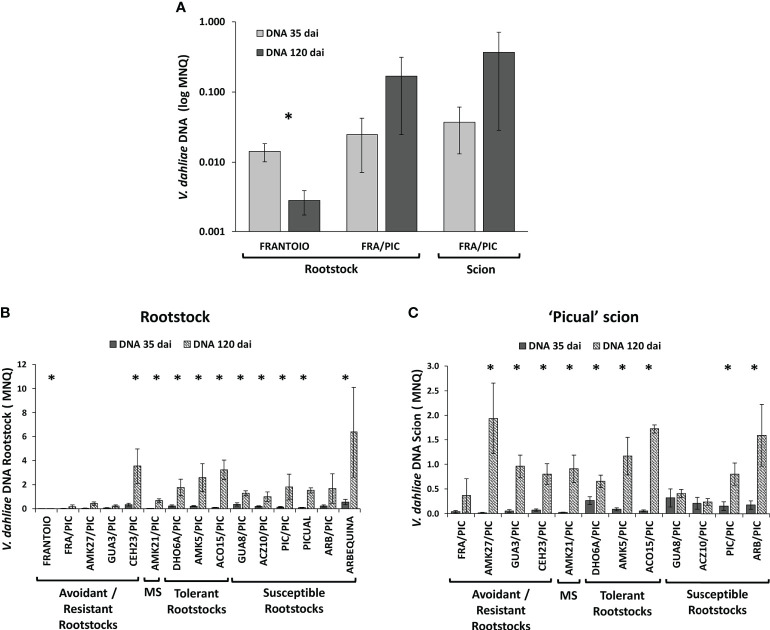
Mean Normalized Quantity (MNQ) of *Verticillium dahliae* DNA in the rootstock trunks of non-grafted ‘Frantoio’ and ‘Frantoio’ grafted with ‘Picual’ (FRA/PIC) and in the corresponding grafted ‘Picual’ scion **(A)**, and in the rootstock trunks **(B)** and ‘Picual’ scions **(C)** of wild olive genotypes grafted with ‘Picual’ at 35 and 120 days after inoculation (dai) with the *V. dahliae* defoliating isolate VD117. Asterisks indicate significant differences between 35 and 120 dai. Results are the mean of 8 plants at 35 dai and 16 plants at 120 dai, and error bars correspond to the standard error.

Wild olive genotypes that present low susceptibility to *V. dahliae* are characterized by controlling the fungus proliferation, so that at a late infection time (120 dai), the content of the fungus is lower than that at an early infection time (35 dai) as we previously reported in Díaz-Rueda et al. (2021). However, when the same low susceptibility genotypes were used as rootstocks of a highly susceptible scion, the proliferation of the fungus did not decrease over time, but rather increased (**Figure 6**). Thus, we could observe that the ungrafted ‘Frantoio’ resistant cultivar reduced the content of *V. dahliae* in the stem at 120 dai, but not when it was used as a rootstock of the grafted ‘Frantoio’ cultivar (**Figure 6A**). All the genotypes used as rootstocks in this work, including those with low or high susceptibility to *V. dahliae*, showed an increase in fungal DNA at 120 dai compared to 35 dai in both plant parts: the rootstock (**Figure 6B**) and the scion (**Figure 6C**).

**Figure 7:**
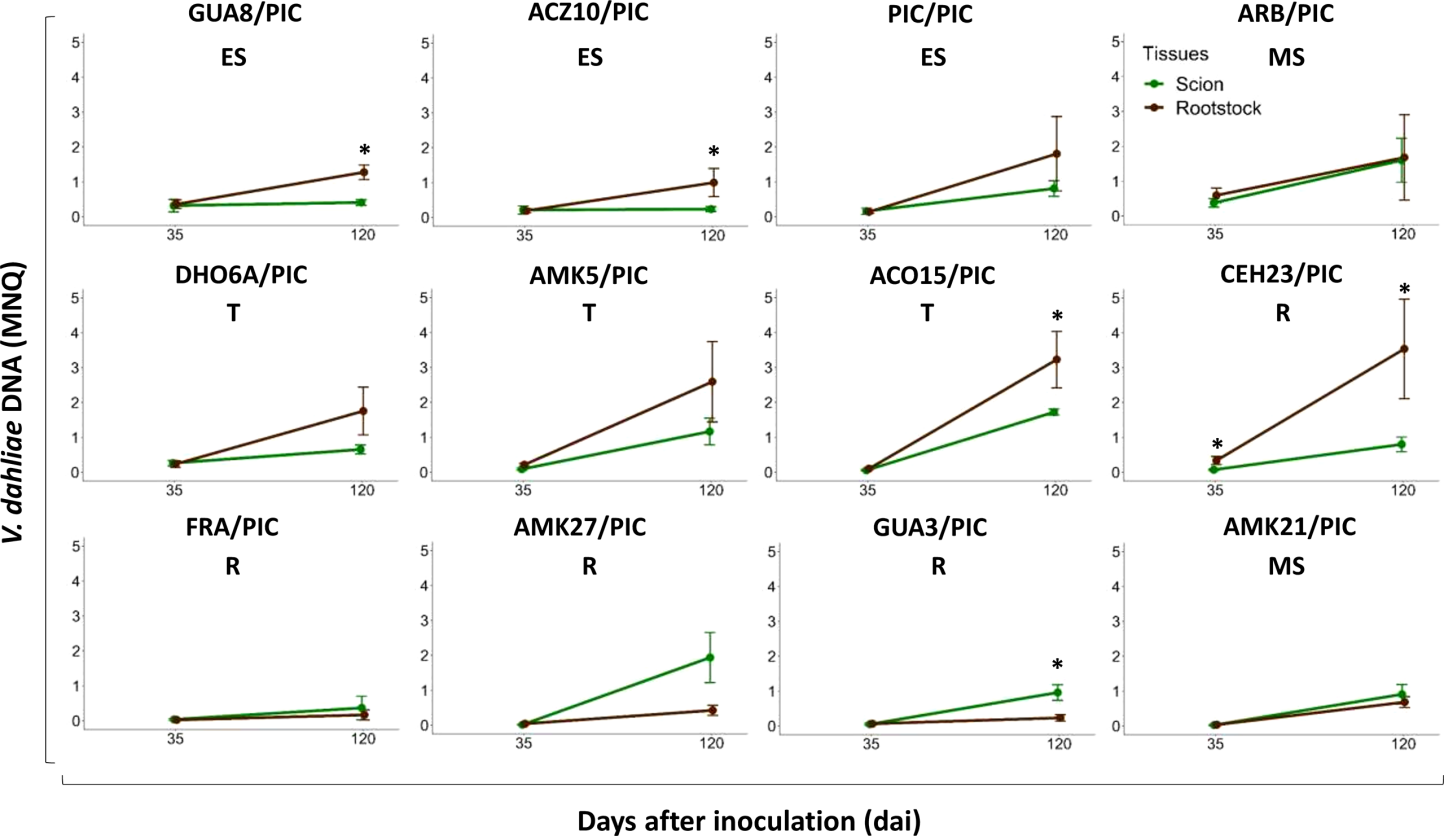
Evolution of *Verticillium dahliae* DNA content in ‘Picual’ scions (green line) and rootstock trunks (brown line) at 35 and 120 days after inoculation (dai) with the defoliating isolate VD117. Points represent the mean of 4 values (one value per block) for each genotype. Error bars represent the standard error of the mean. Asterisks indicate significant differences between different tissues at the same time.

In ‘Picual’ plants grafted with avoidant/resistant rootstocks, *V. dahliae* DNA levels below the graft site (rootstock stem) were lower than those above then graft site (scion stem) after 120 dai (**Figure 7**). However, the opposite was observed in ‘Picual’ plants grafted with tolerant and susceptible rootstocks, which contained more *V. dahliae* DNA below the graft site (rootstock stem) than above then graft site (scion stem) (**Figure 7**). Furthermore, the stem of the tolerant rootstocks CEH23 and ACO15 had higher content of fungal DNA than the stem of the susceptible and extremely susceptible rootstocks (**Figures 6B**, **7**).

**Figure 8:**
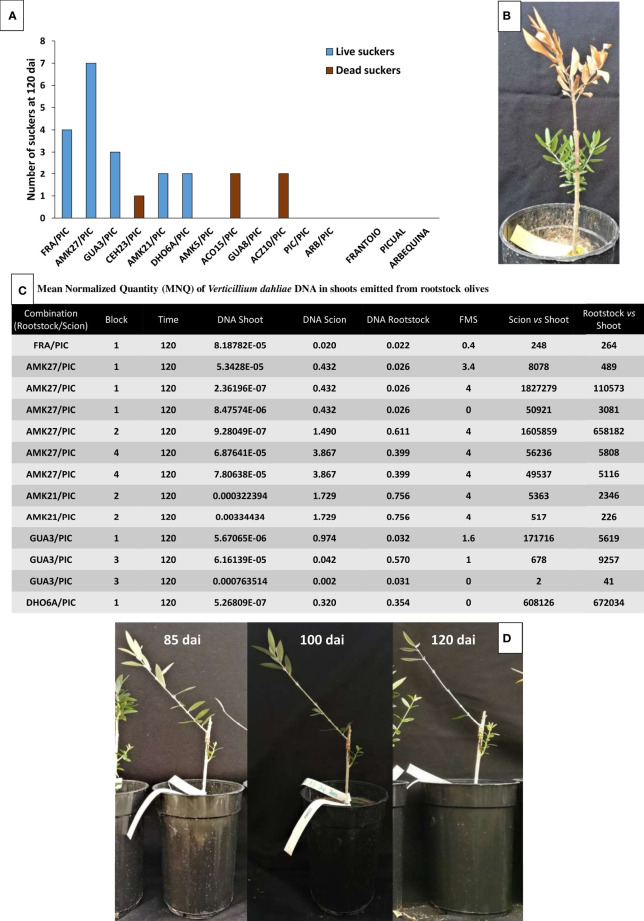
Number of rootstock suckers for each genotype at 120 days after inoculation (dai) (Blue bars correspond to alive suckers and brown bars correspond to dead suckers) **(A)**. Example of an inoculated AMK27 plant at 120 dai showing dead ‘Picual’ scion and asymptomatic suckers grown from the rootstock **(B)**. Mean Normalized Quantity (MNQ) of *Verticillium dahliae* DNA in rootstock suckers at 120 dai with the *V. dahliae* defoliating isolate VD117 **(C)**. Examples of inoculated AMK27/PIC plants at 85, 100 and 120 dai, showing severity of symptoms (defoliation and necrosis) on ‘Picual’ scion and asymptomatic rootstock suckers **(D)**.

The ability of avoidant/resistant rootstocks to better control *V. dahliae* proliferation resulted in the reduction of wilting in the rootstock stem, dealing to frequent sprouting of new shoots that showed no infection symptoms. Interestingly, new sprouts contained around 10^2^ to 10^5^ lower *V. dahliae* levels than the main rootstock stem, regardless of the intensity of symptoms observed in the grafted scion (**Figure 8**). Conversely, sprouting was much less frequent in susceptible rootstocks, and new sprouts turned necrotic within a few days.

## Discussion

The use of resistant rootstocks is one of the most promising and environmentally friendly strategies to control Verticillium wilt of olive. This not only involves the identification of suitable genotypes, but also the study of the interaction between the rootstock and the grafted scion. The resistance of the rootstock can be transmitted to the grafted variety, either by avoiding, limiting or delaying the fungus proliferation, thus impeding the vascular dissemination during the systemic colonization phase, which is the basis of the plant resistance against *V. dahliae* (Pegg and Brady, 2002; Markakis et al., 2009; Gramaje et al., 2013). Control of Verticillium wilt through the use of a resistant rootstock has been shown in this work and was previously reported (Porras Soriano et al., 2003; Bubici and Cirulli, 2012; Yildiz et al., 2020). In fact, resistant wild olive genotypes (Jiménez-Fernández et al., 2016) are being licensed by plant nurseries as olive rootstocks and used for farmers as a control strategy in production fields. In addition, we have also identified a low-susceptibility mechanism based on pathogen tolerance, which means little or no symptoms despite allowing a significant degree of fungus proliferation. Both resistance and tolerance mechanisms have been recently identified in a collection of wild olive germplasm in which several genotypes showed low or very low susceptibility to *V. dahliae* (Díaz-Rueda et al., 2021). If used as rootstocks, we expected that the use of resistant *vs* tolerant genotypes had different effects on a susceptible scion.

The wild genotypes of *Olea europaea* evaluated in this study present a wide genetic variability (Díaz-Rueda et al., 2020), and belong to the subspecies *guanchica* (GUA3 and GUA8), *laperrinei* (DHO6A), *cuspidata* (CEH23) and *europaea* (ACO15, AMK5, AMK21, AMK27 and ACZ10). Other studies have evaluated the susceptibility to *V. dahliae* in wild genotypes of the subspecies *guanchica*, indicating to be a source of resistance genes (Arias-Calderón et al., 2015c); of the subsp. *cuspidata*, considered as a powerful transmitter of resistance (Trapero et al., 2015); and the subsp. *europaea* (Jiménez-Fernández et al., 2016). In this study we have also evaluated the use of wild genotypes of the subspecies *laperrinei, guanchica* and *cuspidata* as rootstocks for the control of Verticillium wilt of olive. All the wild genotypes showed grafting compatibility with the ‘Picual’ scion, even when using genotypes phylogenetically distant to the *europaea* subspecies. No significant differences were observed between the two types of grafting tested within the same rootstock/scion combination, in terms of final symptoms or development of the disease (RAUDPC; **Supplementary Figure S2**). Grafted plants exhibited adequate growth in terms of branch length and number of nodes, demonstrating that the vascular connections were successfully functional.

Non-grafted ‘Frantoio’ and ‘Picual’ reference cultivars used as controls, behaved as resistant and highly susceptible to *V. dahliae*, respectively, as previously reported in controlled conditions (Arias-Calderón et al., 2015a; Arias-Calderón et al., 2015b; Jiménez-Fernández et al., 2016; Sanei and Razavi, 2017; Anguita-Maeso et al., 2021; Díaz-Rueda et al., 2021). However, non-grafted ‘Arbequina’ plants showed high susceptibility to *V. dahliae* in this study (**Figures 1**, **2**). This variety has been previously reported as susceptible (López-Escudero and Mercado-Blanco, 2011; Arias-Calderón et al., 2015a), moderately susceptible (Pérez-Rodríguez et al., 2015; Díaz-Rueda et al., 2021) and even as resistant (Serrano et al., 2021) to the defoliating pathotype of *V. dahliae* in controlled conditions, indicating that ‘Arbequina’ manifests the disease in different ways under different experimental conditions. In this cultivar, we were able to observe less development of symptoms than those corresponding to a high degree of fungus proliferation, indicating certain tolerance properties (**Figure 5A** of Díaz Rueda et al., 2021). Environmental factors such as temperature could cause the degree of proliferation to exceed the tolerance capacity of the cultivar.

‘Picual’ plants grafted onto the reference cultivars (FRA/PIC, ARB/PIC and PIC/PIC) presented a delay in the appearance of symptoms with respect to the non-grafted reference plants at short times (35 dai). This delay in the onset of symptoms has been previously reported in field trials in which the non-grafted ‘Picual’ exhibited disease symptoms earlier than the self-grafted ‘Picual’ (Valverde et al., 2021). The stress caused in the plants by the grafting process may promote vascular occlusion, thus slowing the spread of the pathogen during early infection (Bubici and Cirulli, 2011). This initial resistance was not maintained at 120 dai. ARB/PIC and PIC/PIC plants presented similar symptoms than non-grafted plants. However, when the resistant cultivar ‘Frantoio’ was used as rootstock (FRA/PIC), it reduced the degree of fungus proliferation and infection symptoms in the ‘Picual’ scion.

Not only ‘Frantoio’, but also the resistant genotypes of the wild olive germplasm were more effective than tolerant genotypes in controlling Verticillium wilt when used as rootstocks of the highly susceptible variety ‘Picual’ (GUA3/PIC and AMK27/PIC; **Figures 1**, **2**). Plants grafted on the resistant genotypes (FRA/PIC, GUA3/PIC, AMK27/PIC) and the one that presented a rootstock-scion interaction dealing to a phenotype of higher resistance (AMK21/PIC) were also the ones that showed better growth parameters in relation to the selfgrafted 'Picual' (PIC/PIC) (**Figure 3**). Identification of rootstock-scion combinations with behaviors that are not expected according to the degree of susceptibility previously observed in the non-grafted genotype is of interest in the study of pathogenicity factors in a plant-pathogen interaction. Thus, the moderately susceptible AMK21 (with about 10 times greater fungus proliferation than ‘Frantoio’ in non-grafted plants; **Supplementary Figure S1**; Díaz-Rueda et al., 2021) behaved as a resistant rootstock in ‘Picual’-grafted plants. Conversely, the resistant genotype CEH23, which was highly efficient in reducing *V. dahliae* proliferation (to one third of that of ‘Frantoio’ in non-grafted plants; **Supplementary Figure S1**; Díaz-Rueda et al., 2021) behaved as a susceptible rootstock in ‘Picual’-grafted plants. Therefore, the degree of susceptibility to Verticillium wilt of an olive variety does not always predict its performance as a rootstock. In this way, and looking for a way to predict the degree of susceptibility of an olive genotype, some studies have revealed a relationship between functional traits of olive cultivars and the tolerance to *V. dahliae* (Cardoni et al., 2021), being the root system architecture a relevant difference between susceptible and non-susceptible cultivars. In addition, a different gene expression pattern has been detected in roots of highly resistant and extremely susceptible cultivars: susceptible cultivars have higher expression of genes related to root growth and development, while resistant cultivars show a higher expression of genes related to the defense against pathogens and protein turnover (Ramírez-Tejero et al., 2021). In addition, and searching for a long-term disease control, some resistant olive genotypes have demonstrated a stable resistance when coinfecting the defoliating pathotype of *V. dahliae* with other olive pathogens, such as plant-parasitic nematodes (Palomares-Rius et al., 2016).

We show in this work that tolerant genotypes were as ineffective as the susceptible or extremely susceptible ones in controlling Verticillium wilt. We propose that tolerant rootstocks are less effective in controlling the disease because they allow greater proliferation of the fungus than resistant rootstocks. Once the fungus reaches the stem of the susceptible scion, it spreads rapidly. We could even quantify significantly higher amounts of fungus in the ‘Picual’ scion grafted on the tolerant rootstocks compared to the susceptible ones at 120 dai (**Figure 7**). But this was probably a consequence of the fact that the scion grafted on susceptible or very susceptible rootstocks were dead for a longer time, which may reduce the amount of live fungus.

Passage of the infection to the susceptible scion also occurred in plants grafted onto resistant genotypes, but later. Although the rootstocks are able to partially control Verticillium wilt according to their degree of susceptibility to *V. dahliae*, our results show that the ability to control the infection was not adequately transferred to a highly susceptible grafted scion. The infection of the susceptible scion worsens in turn the resistance or tolerance capacity of the rootstock, even though it is of low susceptibility (**Figures 6**, **7**). Therefore, the ability to control Verticillium wilt exhibited by ‘Frantoio’ and other resistant cultivars can be overcome, not only when used as rootstock of a susceptible scion, as also shown by Valverde et al. (2021), but also under moderate and high fungal inoculum density under long-term field conditions (Trapero et al., 2013) and under different irrigation systems (Pérez-Rodríguez et al., 2015).

Interestingly, adventitious shoots emerged from resistant rootstocks (‘Frantoio’, AMK27, GUA3 and AMK21) showed an asymptomatic behavior with little or no presence of the pathogen (**Figure 8**). It has been recently reported that the size of the xylem vessels is related to the resistance of plants to pathogens (Pouzoulet et al., 2017). New emerging shoots probably present narrower vessels that could be less exposed to fungal proliferation. In fact, the new shoots emerged from the tolerant and susceptible rootstocks had higher mortality (**Figure 8A**), indicating that their greater susceptibility to Verticillium wilt could be a direct consequence of a larger size of their vascular bundles, compared to the resistant genotypes.

Some aspects should be considered in this study that could explain the high incidence of the disease even in plants grafted onto resistant rootstocks: i) a higher incidence of the disease due to environmental factors, as previously stressed, which made even ‘Arbequina’ plants behaved as extremely susceptible to *V. dahliae;* ii) the high inoculum density used in the inoculation tests (10^7^ microsclerotia/mL of soil), far from the inoculum densities found in a real scenario, with 5-21 microsclerotia/g of soil in infested plots under field conditions (Trapero et al., 2015; Valverde et al., 2021); iii) the young plant material of the wild genotypes, which is more susceptible to infection, xylem cavitation and embolism than mature plants; iv) *in-vitro* micropropagated plants may impair the composition of the xylem microbiome being more susceptible than those obtained by conventional nursery multiplication (Anguita-Maeso et al., 2021). Resistant rootstocks have been successfully applied to control *V. dahliae* in different herbaceous species as watermelon (Devi et al., 2021), eggplant (Attavar and Miles, 2021) and tomato (Papadaki et al., 2017), and also in different woody species such as avocado (Haberman et al., 2020) and pistachio (Epstein et al., 2004). Therefore, we may expect that avoidant/resistant genotypes could be more effective in controlling Verticillium wilt under normal field conditions, with lower inoculum densities and using mature plant material. The evaluation of new olive material from different germplasm collections (Arias-Calderón et al., 2015b; Trapero et al., 2015; Díaz-Rueda et al., 2021), could provide rootstocks capable of effectively controlling olive Verticillium wilt.

## Conclusion

In conclusion, our results confirmed that: the degree of susceptibility to Verticillium wilt of an olive variety does not predict its performance as a rootstock; to use a very low susceptible genotype as rootstock of a susceptible scion increases the susceptibility of the genotype used as rootstock; in any case, avoidant/resistant rootstocks are more effective than tolerant rootstocks in reducing the susceptibility of the grafted plant to *V. dahliae*.

## Data availability statement

The original contributions presented in the study are included in the article/**Supplementary Material**. Further inquiries can be directed to the corresponding authors.

## Author contributions

PD-R participated in all experimental tasks, particularly in plant material production, DNA quantification by qPCR and data analysis, as well as in writing the manuscript; PP-T and AA contributed in the inoculation of the plants and participated in data analysis; FD-G, PA-R contributed in the inoculation of the plants and DNA quantification by qPCR; NC designed the experiment, participated in inoculation of the plants and wrote the article; JC-F conceived the project, obtained the funds for its financing, supervised the experiment and the manuscript. All authors contributed to the article and approved the submitted version.

## Funding

This work has been funded through the RECUPERA-2020 project (Ref. 20134R089), the IFAPA contract CAICEM 15-02 and the Spanish National Research Council ‘Proyectos Intramurales’ CSIC-201640E069, CSIC-201740E041, CSIC-201940E077 and CSIC-202040E057.

## Acknowledgments

We thank Viveros Sevilla S.L. for supplying plant material and for the use of their greenhouse facilities. Authors are especially grateful for the technical assistance of Isidro Álvarez and David Romero.

## Conflict of interest

The authors declare that the research was conducted in the absence of any commercial or financial relationships that could be construed as a potential conflict of interest.

## Publisher’s note

All claims expressed in this article are solely those of the authors and do not necessarily represent those of their affiliated organizations, or those of the publisher, the editors and the reviewers. Any product that may be evaluated in this article, or claim that may be made by its manufacturer, is not guaranteed or endorsed by the publisher.
