# Supplementary material for: Avoidant/resistant rather than tolerant olive rootstocks are more effective in controlling Verticillium wilt
Source: Front Plant Sci. 2022 Oct 17;13:1032489. doi: 10.3389/fpls.2022.1032489 (PMC9619059; doi:10.3389/fpls.2022.1032489)
Supplement: Supplementary file 1 [file Presentation_1.pptx]

## Slide 1
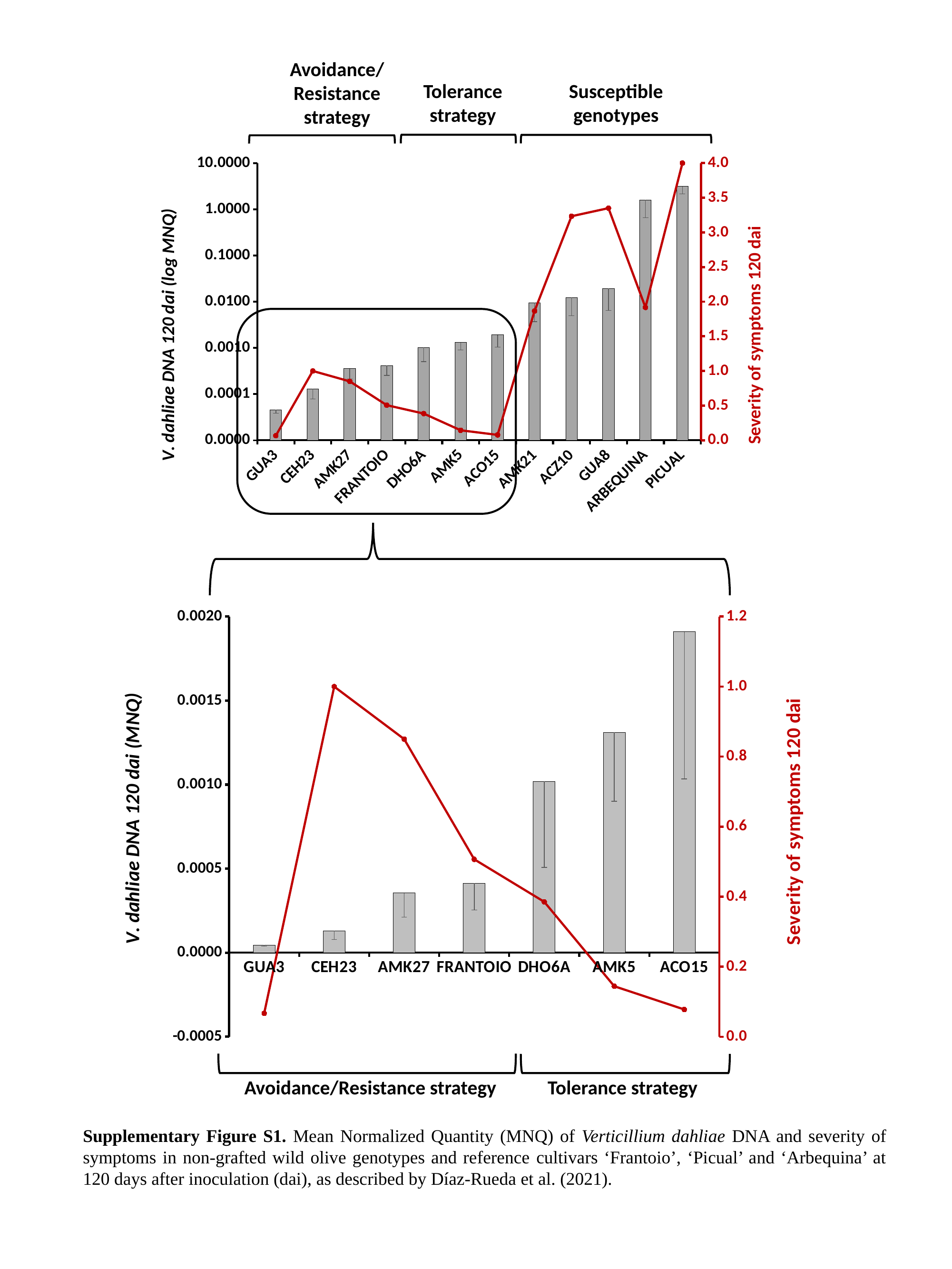

Avoidance/Resistance strategy
Tolerance strategy
Susceptible genotypes
### Chart
| Category | MNQ 120 dai | SYMTOMS 120 dai (FMS) |
|---|---|---|
| GUA3 | 4.5778375248286364e-05 | 0.06666666666666667 |
| CEH23 | 0.0001300527573507737 | 1.0 |
| AMK27 | 0.0003553301259141112 | 0.85 |
| FRANTOIO | 0.0004129544271685373 | 0.5066666666666665 |
| DHO6A | 0.0010193777797400919 | 0.3852777777777778 |
| AMK5 | 0.001310889681177435 | 0.1441666666666666 |
| ACO15 | 0.0019092039553397003 | 0.07777777777777782 |
| AMK21 | 0.009539490883644374 | 1.866666666666665 |
| ACZ10 | 0.01236438196997708 | 3.233333333333333 |
| GUA8 | 0.01934826706289889 | 3.35 |
| ARBEQUINA | 1.594417493878252 | 1.9181249999999999 |
| PICUAL | 3.1309325931876177 | 4.0 |
### Chart
| Category | | SYMTOMS 120 dai (FMS) |
|---|---|---|
| GUA3 | 4.5778375248286364e-05 | 0.06666666666666667 |
| CEH23 | 0.0001300527573507737 | 1.0 |
| AMK27 | 0.0003553301259141112 | 0.85 |
| FRANTOIO | 0.0004129544271685373 | 0.5066666666666665 |
| DHO6A | 0.0010193777797400919 | 0.3852777777777778 |
| AMK5 | 0.001310889681177435 | 0.1441666666666666 |
| ACO15 | 0.0019092039553397003 | 0.07777777777777782 |
Avoidance/Resistance strategy
Tolerance strategy
Supplementary Figure S1. Mean Normalized Quantity (MNQ) of Verticillium dahliae DNA and severity of symptoms in non-grafted wild olive genotypes and reference cultivars ‘Frantoio’, ‘Picual’ and ‘Arbequina’ at 120 days after inoculation (dai), as described by Díaz-Rueda et al. (2021).

## Slide 2
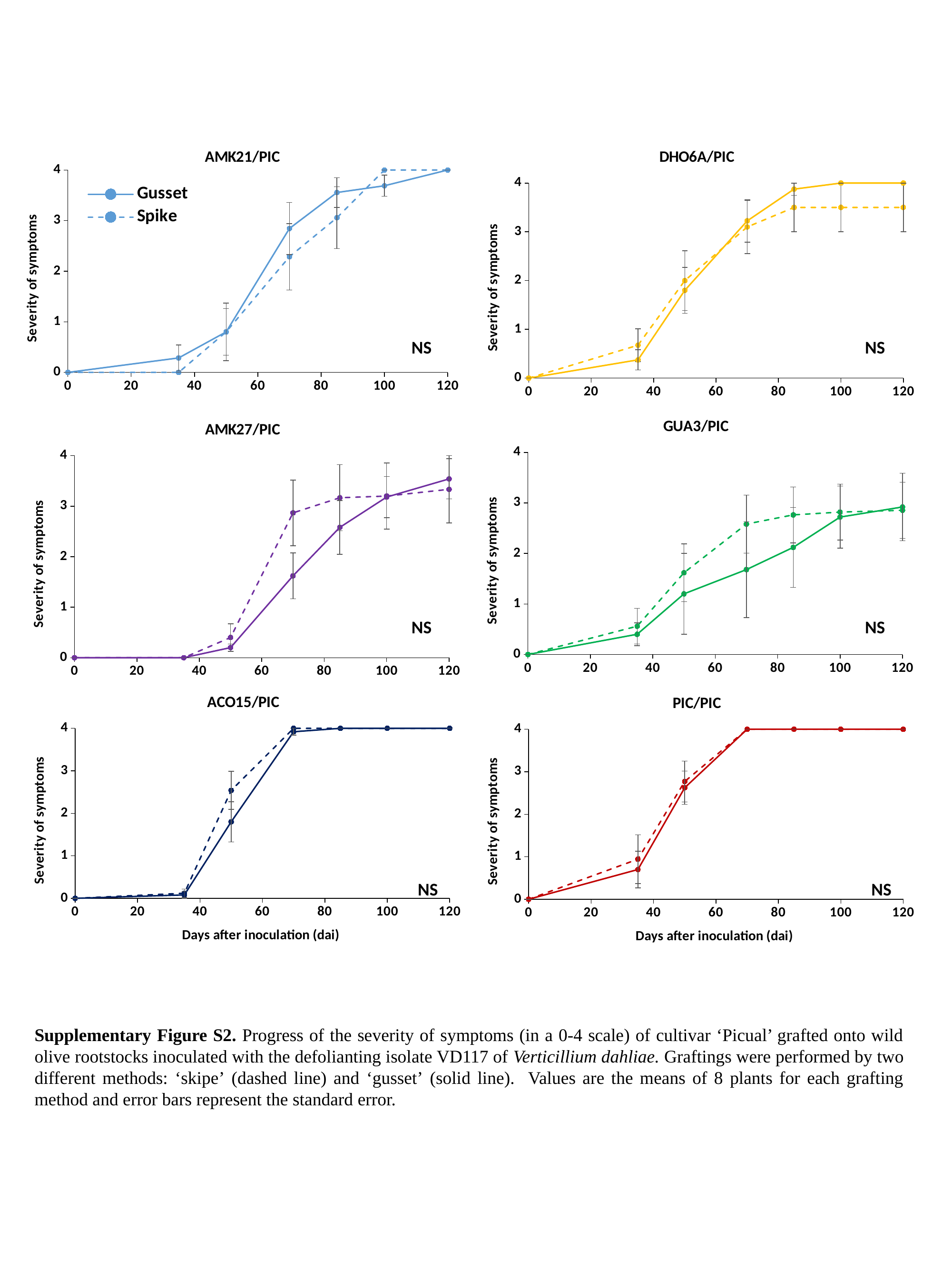

### Chart: AMK21/PIC
| Category | Gusset | Spike |
|---|---|---|
### Chart: DHO6A/PIC
| Category | DHO6A-gusset | DHO6A-spike |
|---|---|---|NS
NS
### Chart: GUA3/PIC
| Category | GUA3-gusset | GUA3-spike |
|---|---|---|
### Chart: AMK27/PIC
| Category | AMK27-gusset | AMK27-spike |
|---|---|---|NS
NS
### Chart: ACO15/PIC
| Category | ACO15-gusset | ACO15-spike |
|---|---|---|
### Chart: PIC/PIC
| Category | PICUAL-gusset | PICUAL-spike |
|---|---|---|NS
NS
Supplementary Figure S2. Progress of the severity of symptoms (in a 0-4 scale) of cultivar ‘Picual’ grafted onto wild olive rootstocks inoculated with the defolianting isolate VD117 of Verticillium dahliae. Graftings were performed by two different methods: ‘skipe’ (dashed line) and ‘gusset’ (solid line). Values are the means of 8 plants for each grafting method and error bars represent the standard error.

## Slide 3
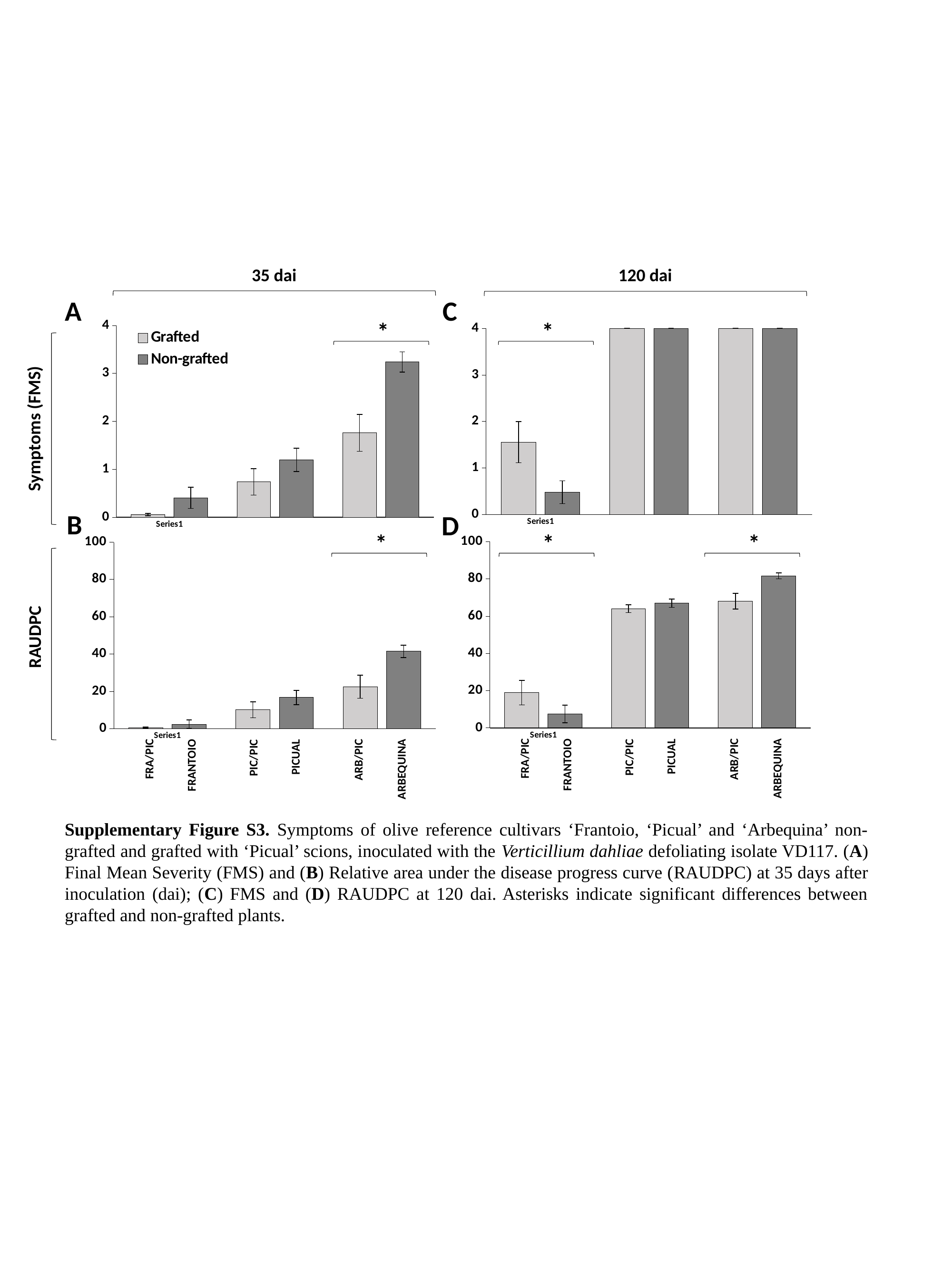

35 dai
120 dai
A
C
*
*
### Chart
| Category | Grafted | Non-grafted |
|---|---|---|
| | 0.05818181818181818 | 0.4071428571428571 |
| | 0.7391304347826086 | 1.1999999999999997 |
| | 1.7619047619047619 | 3.2399999999999998 |
### Chart
| Category | Grafted | Non-grafted |
|---|---|---|
| | 1.5571428571428572 | 0.48181818181818165 |
| | 4.0 | 4.0 |
| | 4.0 | 4.0 |
Symptoms (FMS)
B
D
*
*
*
### Chart
| Category | Grafted | Non-grafted |
|---|---|---|
| | 18.936011904761905 | 7.476325757575757 |
| | 64.05555555555556 | 66.96875 |
| | 68.09722222222223 | 81.61458333333336 |
### Chart
| Category | Grafted | Non-grafted |
|---|---|---|
| | 0.5357142857142857 | 2.3863636363636362 |
| | 10.166666666666666 | 16.75 |
| | 22.5 | 41.458333333333336 |
RAUDPC
FRA/PIC
ARB/PIC
ARBEQUINA
FRANTOIO
PICUAL
FRA/PIC
ARB/PIC
ARBEQUINA
PIC/PIC
FRANTOIO
PICUAL
PIC/PIC
Supplementary Figure S3. Symptoms of olive reference cultivars ‘Frantoio, ‘Picual’ and ‘Arbequina’ non-grafted and grafted with ‘Picual’ scions, inoculated with the Verticillium dahliae defoliating isolate VD117. (A) Final Mean Severity (FMS) and (B) Relative area under the disease progress curve (RAUDPC) at 35 days after inoculation (dai); (C) FMS and (D) RAUDPC at 120 dai. Asterisks indicate significant differences between grafted and non-grafted plants.

## Slide 4
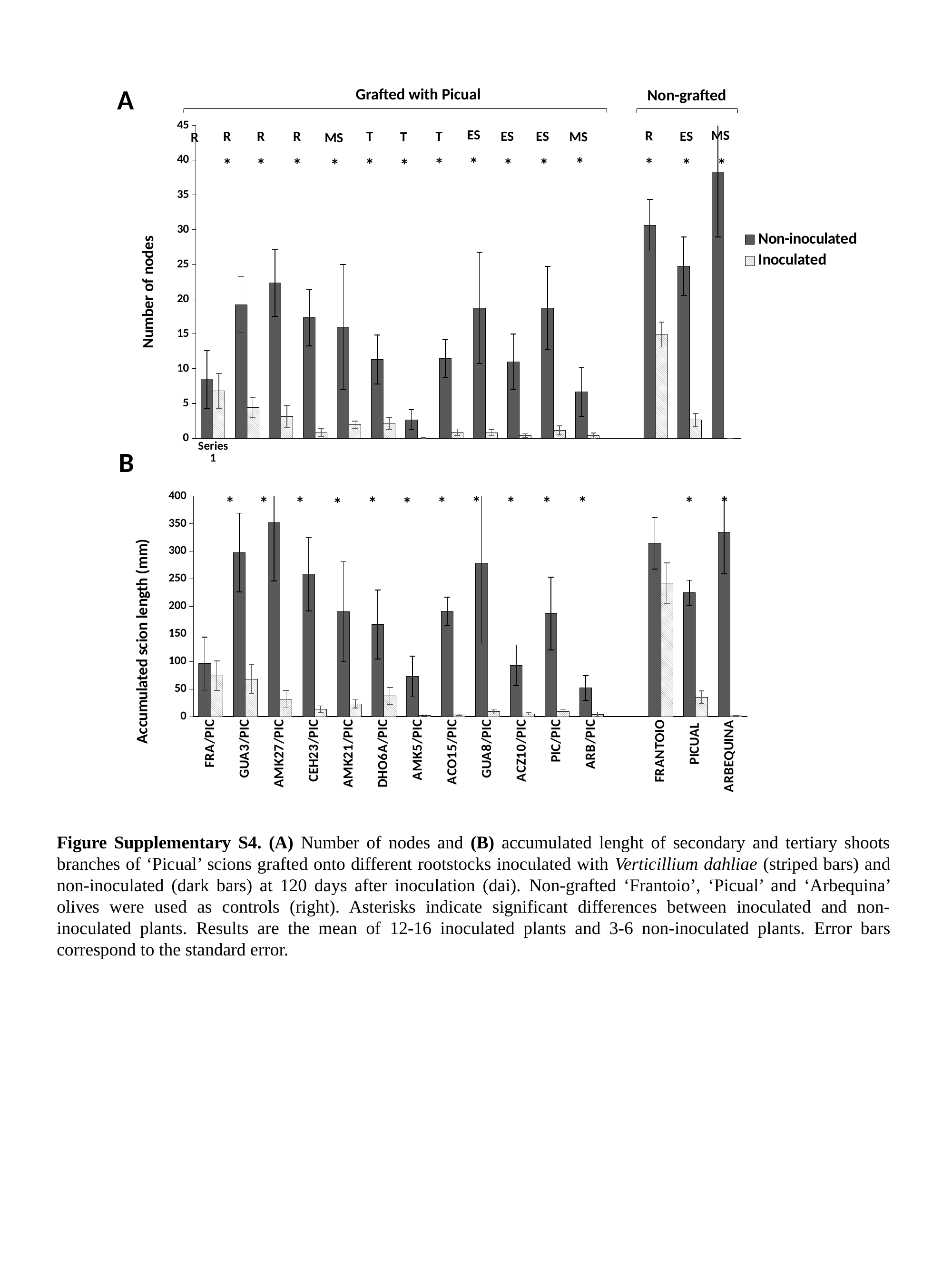

A
Grafted with Picual
Non-grafted
### Chart
| Category | Non-inoculated | Inoculated |
|---|---|---|
| | 8.5 | 6.785714285714286 |
| | 19.2 | 4.4375 |
| | 22.333333333333332 | 3.1333333333333333 |
| | 17.333333333333332 | 0.8125 |
| | 16.0 | 1.9375 |
| | 11.333333333333334 | 2.125 |
| | 2.6666666666666665 | 0.0625 |
| | 11.5 | 0.8666666666666667 |
| | 18.75 | 0.8 |
| | 11.0 | 0.35714285714285715 |
| | 18.75 | 1.1333333333333333 |
| | 6.666666666666667 | 0.38461538461538464 |
| | None | None |
| | 30.666666666666668 | 14.909090909090908 |
| | 24.75 | 2.6 |
| | 38.333333333333336 | 0.0 |ES
MS
R
T
ES
R
R
R
T
ES
ES
T
MS
R
MS
*
*
*
*
*
*
*
*
*
*
*
*
*
*
B
### Chart
| Category | Non-inoculated | Inoculated |
|---|---|---|
| FRA/PIC | 96.0 | 74.28571428571429 |
| GUA3/PIC | 297.8 | 67.9375 |
| AMK27/PIC | 352.3333333333333 | 31.8 |
| CEH23/PIC | 258.5 | 13.375 |
| AMK21/PIC | 190.5 | 23.125 |
| DHO6A/PIC | 167.33333333333334 | 37.3125 |
| AMK5/PIC | 73.0 | 2.0625 |
| ACO15/PIC | 191.5 | 3.2666666666666666 |
| GUA8/PIC | 278.25 | 9.133333333333333 |
| ACZ10/PIC | 93.0 | 5.214285714285714 |
| PIC/PIC | 187.0 | 8.8 |
| ARB/PIC | 52.0 | 4.230769230769231 |
| | None | None |
| FRANTOIO | 314.6666666666667 | 241.86363636363637 |
| PICUAL | 224.75 | 35.05 |
| ARBEQUINA | 334.6666666666667 | 1.0833333333333333 |*
*
*
*
*
*
*
*
*
*
*
*
*
Figure Supplementary S4. (A) Number of nodes and (B) accumulated lenght of secondary and tertiary shoots branches of ‘Picual’ scions grafted onto different rootstocks inoculated with Verticillium dahliae (striped bars) and non-inoculated (dark bars) at 120 days after inoculation (dai). Non-grafted ‘Frantoio’, ‘Picual’ and ‘Arbequina’ olives were used as controls (right). Asterisks indicate significant differences between inoculated and non-inoculated plants. Results are the mean of 12-16 inoculated plants and 3-6 non-inoculated plants. Error bars correspond to the standard error.
